# Redox-induced structural changes in the di-iron and di-manganese forms of *Bacillus anthracis* ribonucleotide reductase subunit NrdF suggest a mechanism for gating of radical access

**DOI:** 10.1007/s00775-019-01703-z

**Published:** 2019-08-13

**Authors:** Kristīne Grāve, Wietske Lambert, Gustav Berggren, Julia J. Griese, Matthew D. Bennett, Derek T. Logan, Martin Högbom

**Affiliations:** 1 Department of Biochemistry and Biophysics, Stockholm University, Svante Arrhenius väg 16C, 10691 Stockholm, Sweden; 2 PRA Health Sciences, Amerikaweg 18, 9407 TK Assen, The Netherlands; 3 Department of Chemistry, Ångström Laboratory, Uppsala University, Lägerhyddsvägen 1, 75120 Uppsala, Sweden; 4 Department of Cell and Molecular Biology, Uppsala University. BMC, Box 596, 75124 Uppsala, Sweden; 5 Department of Biochemistry and Structural Biology, Lund University, Box 124, 221 00 Lund, Sweden

**Keywords:** Oxidoreductase, Metalloprotein, Carboxylate shift, X-ray crystallography, Ferritin superfamily

## Abstract

Class Ib ribonucleotide reductases (RNR) utilize a di-nuclear manganese or iron cofactor for reduction of superoxide or molecular oxygen, respectively. This generates a stable tyrosyl radical (Y·) in the R2 subunit (NrdF), which is further used for ribonucleotide reduction in the R1 subunit of RNR. Here, we report high-resolution crystal structures of *Bacillus anthracis* NrdF in the metal-free form (1.51 Å) and in complex with manganese (Mn^II^/Mn^II^, 1.30 Å). We also report three structures of the protein in complex with iron, either prepared anaerobically (Fe^II^/Fe^II^ form, 1.32 Å), or prepared aerobically in the photo-reduced Fe^II^/Fe^II^ form (1.63 Å) and with the partially oxidized metallo-cofactor (1.46 Å). The structures reveal significant conformational dynamics, likely to be associated with the generation, stabilization, and transfer of the radical to the R1 subunit. Based on observed redox-dependent structural changes, we propose that the passage for the superoxide, linking the FMN cofactor of NrdI and the metal site in NrdF, is closed upon metal oxidation, blocking access to the metal and radical sites. In addition, we describe the structural mechanics likely to be involved in this process.

**Electronic supplementary material:**

The online version of this article (10.1007/s00775-019-01703-z) contains supplementary material, which is available to authorized users.

## Introduction

Ribonucleotide reductase (RNR) is an essential enzyme in all domains of life. It catalyzes ribonucleotide reduction to the corresponding deoxyribonucleotides, which are further used in DNA synthesis and repair [1]. RNRs are complex enzymes that utilize radical chemistry for their catalytic activity. RNRs are grouped into classes I, II, and III (reviewed in [2, 3]). Class I RNRs are oxygen-dependent and employ a radical-generating subunit denoted β or R2. After generation, the radical is transferred in excess of 35 Å to the active site in the catalytic subunit, denoted α or R1, generating a cysteine thiyl radical that initiates ribonucleotide reduction. With the exception of the recently discovered metal-free R2 subunit in common human pathogens [4, 5], R2 proteins house a di-metal cofactor responsible for generation and sometimes storage of the radical. The nature of the radical species and metal site differs between the R2 subunits; thus, class I RNRs are further subdivided into five subclasses (Ia–e): Ia (Fe^III^/Fe^III^- tyrosyl radical), Ib (Mn^III^/Mn^III^-tyrosyl radical), Ic (Mn^IV^/Fe^III^), and (Mn^IV^/Mn^III^) in the recently proposed class Id [2, 3, 6–8]. The metal-free R2, denoted class Ie, requires the flavoprotein NrdI and molecular oxygen for generation of a stable 3,4-dihydroxyphenylalanine (DOPA) radical [4].

Many organisms have been found to utilize more than one class of RNR, most likely depending on their ecological niche and lifecycle [9]. Similarly, *Bacillus anthracis* (*Ba*), a Gram-positive zoonotic spore-forming bacterium and the causative agent of anthrax, encodes two types of RNRs belonging to classes III and Ib. In contrast to the obligate anaerobic class III enzyme, the class Ib RNR is active during aerobiosis [3], i.e., the active stages of anthrax, and thus may represent an attractive drug target. Interestingly, the di-manganese metallo-cofactor of class Ib R2 (NrdF) is unreactive towards molecular oxygen and requires an activase, the flavoprotein NrdI, to provide superoxide as the oxidant of the metal site [10]. The NrdF–NrdI complex forms a channel, presumed to facilitate superoxide delivery to the Mn_2_^II/II^ metallo-cofactor [11–13]. Reaction with superoxide generates a high-valent Mn_2_^III/IV^ intermediate state which decays to produce the tyrosyl radical (Y·) and an Mn_2_^III/III^ metal site [10]. While the di-manganese cofactor appears to be the physiologically relevant form of class Ib R2 proteins, they can also function using a di-iron site via direct oxidation by molecular oxygen, resulting in an Fe_2_^III/III^ metal site and Y· [11, 14].

Here, we report several high-resolution crystal structures of *B. anthracis* NrdF in the metal-free (apo) form, as well as the Fe- and Mn-containing forms in the reduced state, prepared for reaction with molecular oxygen or superoxide, respectively. We also report the structure of a partially oxidized Fe-containing protein after reaction with molecular oxygen. The latter structure reveals dynamics of a helical segment proximal to the metal site. Such dynamics are a characteristic feature of the oxygen-dependent R2 proteins and we hypothesize that they play an important role in tyrosyl radical turnover and stability. Considering that *B. anthracis* poses a risk of being used as a biological weapon, the structures may serve as a basis for drug design against this pathogen.

## Materials and methods

### Materials

*Escherichia coli* strains DH5α (Stratagene) and Rosetta (DE3) (Novagen) were used for cloning and protein expression, respectively. Wild-type *B. anthracis* STERNE 7700 pXO1^−^/pXO2^−^ (lacking both virulence plasmids) was obtained from the Swedish Defence Research Agency. *E. coli* strains were routinely grown in LB medium at 37 °C, and *B. anthracis* was grown in Brain Heart Infusion medium (Becton–Dickinson) at 37 °C. When required, antibiotics and chromogenic substrates were added at the following concentrations: ampicillin 50 µg/mL, chloramphenicol 20 µg/mL, kanamycin 50 µg/mL, and 5-bromo-4-chloro-3-indolyl-β-d-galactopyranoside (X-Gal) 30 µg/mL. Genomic DNA from *B. anthracis* was extracted using the DNeasy tissue kit (Qiagen) according to the manufacturer’s instructions.

### Cloning of *B. anthracis* NrdF, protein expression and purification

The *nrdF*-coding sequence (UniProt: Q81TB4) was amplified by PCR using the genomic DNA of *B. anthracis* STERNE 7700 and the following primers: BaNrdF_For, 5′-ATACATATGCGTGCGGTAAACTGG-3′, and BaNrdF_Rev 5′-ATAAAGCTTAAAAATTAAACACGAAGTCATC -3′, introducing NdeI and HindIII restriction sites (underlined) at the start and at the end of the amplification product, respectively. The purified PCR product was cloned into plasmid pGEM-T easy (Promega), giving the plasmid pETS137. After digestion with NdeI and HindIII (Fermentas), the *nrdF* fragment was ligated into pET22b (Novagen), resulting in a non-tagged full-length construct in pETS145. The pETS145 plasmid was further transformed into *E. coli* Rosetta (DE3) for protein expression. *Ba* NrdF was purified as previously described (Ref. [15]) with two additional steps after Q-Sepharose chromatography. Particularly, Q-Sepharose protein fractions containing *Ba* NrdF were incubated with 10 mM EDTA and 2.5 mM sodium dithionite on ice for 1.5 h and re-chromatographed on a HiLoad Superdex 200 16/60 pg (GE Healthcare) at a rate of 1 mL/min in 20 mM Tris–HCl pH 7.6 and 100 mM KCl. *Ba* NrdF protein fractions were checked by SDS-PAGE, pooled, and concentrated to 30 mg/mL using Centriprep-30 tubes (Millipore), flash cooled in liquid N_2_, and stored at − 80 °C until further use.

### Total reflection X-ray fluorescence spectrometry (TXRF)

Metal contents of the apoprotein preparation were quantified using TXRF analysis on a Bruker PicoFox instrument [16]. A gallium standard (Sigma) was added to duplicate samples (v/v 1:1) prior to the measurements. TXRF spectra were analyzed using the routines provided with the spectrometer.

### Crystallization and crystallographic data collection

Apo *Ba* NrdF was crystallized in hanging drop vapor diffusion experiments. Drops containing 1.5 μL of protein at 10–12 mg/mL in 0.02 M Tris buffer pH 7.6 and 0.1 M KCl were mixed with equal volumes of reservoir solution containing 1.8 M or 2.0 M ammonium sulphate and 0.1 M Bis–Tris methane buffer, pH 6.5, at 20 °C. Crystals grew within a few days. Larger crystals suitable for data collection, measuring approximately 0.2 × 0.1 × 0.1 mm, were produced by streak seeding of fresh drops containing 1.5 μL of protein at 4–6 mg/mL and 2–3 μL of reservoir solution. Crystals were cryo-protected by transferring briefly to a solution of 3.4 M sodium malonate, pH 6.5, and flash cooled in liquid N_2_ prior to data collection.

Apo *Ba* NrdF crystals were soaked in air-saturated mother liquor additionally containing freshly prepared 5 mM MnCl_2_ or (NH_4_)_2_Fe(SO_4_)_2_ for 20–30 min to obtain metal-bound crystals. In the presence of O_2_ and Fe^II^, the metal center formed should activate, leading to generation of the Y·, while the Mn-bound form is not expected to react with O_2_. These crystals were briefly washed in 3.4 M sodium malonate, pH 6.5, and flash cooled in liquid nitrogen prior to data collection. To obtain the non-activated reduced di-iron cofactor, apo *Ba* NrdF crystals were soaked in mother liquor additionally containing 5 mM (NH_4_)_2_Fe(SO_4_)_2_, 0.5% (w/v) sodium dithionite, 0.5 mM phenosafranin, and 25% glycerol (v/v) for 1 h and flash cooled in liquid nitrogen prior to data collection [17]. A preliminary *Ba* NrdF diffraction data set was collected at − 173 °C at I911-3/MAX II (Lund, Sweden). Diffraction data presented here were collected at − 173 °C at PX14.1/BESSY (Helmholtz Center Berlin, Germany), X06SA, and X06DA/SLS (Villigen, Switzerland).

### Structure determination, model building, and refinement

Crystals of *Ba* NrdF belong to space group P2_1_, with cell dimensions as shown in Table 1. The asymmetric unit contains one NrdF dimer, giving a Matthews coefficient [18, 19] of 2.13 Å^3^/Da and a solvent content of 42.3%. Data were processed using XDS [20] and programs from the CCP4 suite [21, 22].

**Table 1:** Data collection and refinement statistics

	Apo	Fe_2_-aer	Fe_2_-anox	Mn_2_	Fe_2_-semiox
PDB ID	6QO5	6QO7	6QO8	6QO9	6QOB
Data collection statistics					
Wavelength (Å)	0.92	0.92	0.92	0.92	1.00
Resolution range (Å)	40.35–1.51 (1.57–1.51)	41.82–1.63 (1.69–1.63)	42.26–1.32 (1.37–1.32)	27.58–1.30 (1.35–1.30)	45.93–1.46 (1.51–1.46)
Space group	P 2_1_	P 2_1_	P 2_1_	P 2_1_	P 2_1_
Unit cell dimensions a, b, c (Å)	57.19, 59.81, 95.23	57.29, 59.96, 95.36	57.56, 61.46, 95.79	57.53, 61.23, 95.84	57.14, 60.38, 95.76
Unit cell dimensions *β* (°)	107.08	107.11	106.59	106.48	106.38
Unique reflections	95,740 (9403)	77,058 (7581)	147,577 (13,178)	155,351 (15,233)	107,862 (9731)
Multiplicity	3.7 (3.8)	3.8 (3.8)	3.2 (2.3)	3.3 (3.3)	3.4 (3.0)
Completeness (%)	99.46 (97.94)	99.61 (98.35)	97.86 (87.89)	99.00 (97.65)	98.67 (89.12)
Mean I/sigma (I)	16.35 (2.11)	12.80 (2.05)	14.70 (1.75)	14.90 (2.16)	11.48 (1.62)
Wilson B factor (Å^2^)	18.59	19.44	12.83	10.77	15.61
R-merge	0.044 (0.622)	0.062 (0.628)	0.040 (0.437)	0.048 (0.532)	0.058 (0.584)
R-meas	0.051 (0.723)	0.072 (0.729)	0.048 (0.559)	0.057 (0.632)	0.069 (0.710)
R-pim (I)	0.0261 (0.365)	0.036 (0.366)	0.026 (0.343)	0.031 (0.338)	0.037 (0.399)
CC_1/2_	0.999 (0.805)	0.999 (0.778)	0.999 (0.817)	0.999 (0.796)	0.999 (0.746)
Refinement statistics					
Reflections used in refinement	95,758 (9403)	77,191 (7581)	147,262 (13,165)	155,158 (15,228)	107,820 (9731)
Reflections used for R-free	4788 (470)	3860 (379)	7360 (657)	7756 (761)	5391 (486)
R-work	0.17 (0.27)	0.16 (0.28)	0.13(0.21)	0.14 (0.19)	0.17 (0.28)
R-free	0.19 (0.30)	0.19 (0.30)	0.16 (0.24)	0.16 (0.21)	0.19 (0.28)
Protein residues	580	579	574	576	575
Number of non-hydrogen atoms	5078	5069	5371	5385	5194
Number of water molecules	305	317	546	492	357
Number of metal ions	0	4	4	4	4
RMS, bonds (Å)	0.013	0.013	0.011	0.012	0.012
RMS, angles (°)	1.20	1.18	1.13	1.44	1.17
Ramachandran favored (%)	98.95	99.12	99.12	99.13	98.95
Ramachandran allowed (%)	1.05	0.88	0.88	0.87	1.05
Ramachandran outliers (%)	0.00	0.00	0.00	0.00	0.00
Rotamer outliers (%)	0.39	0.39	0.00	0.19	0.00
Clashscore	1.68	1.90	1.67	1.95	3.95
Average B factor (Å^2^)	24.13	25.36	18.08	16.73	20.22

Statistics for the highest resolution shell are shown in parentheses

*RMS* root mean square

A preliminary structure of the *Ba* NrdF was solved by molecular replacement (MR) using the MR pipeline MrBUMP [23] with Molrep [24] as an MR engine. The dimer of NrdF from *Corynebacterium ammoniagenes*, PDB code 1KGN [25], with 46% sequence identity to *Ba* NrdF, was successful as search model after side-chain truncation using Molrep [26]. After structure refinement in Refmac [27] and rebuilding using Coot [28], it was apparent that the metal site was partially metal-occupied. Thus, an additional de-metallation step during purification was used for the further structural studies reported here.

The metal-free *Ba* NrdF structure was solved by Phaser (Phenix suite) as an MR engine [29, 30] using the partially metal-containing *Ba* NrdF dimer (unpublished). After ensuring that the protein was not metal-contaminated, the apo *Ba* NrdF was used as an MR search model for the metal-containing structures reported here. The models were iteratively rebuilt using Coot [28] and refined using phenix.refine [31]. Refinement generally included bulk solvent corrections, individual atomic coordinate and isotropic *B* factor refinement, and occupancy refinement for alternate conformations. Metal–ligand bond restraints were used in the Fe_2_-semiox structure refinement to facilitate correct placement of alternative metal-chelating ligand conformations and solvent molecules at the metal sites; in the other structures, the metal–ligand bonds were not restrained. In the Fe_2_-aer and Mn_2_*Ba* NrdF structures, anisotropic *B* factors were refined (including metal ions) due to the sufficiently high resolution of the data (1.32 and 1.30 Å, respectively). Solvent molecules were added with phenix.refine and manually. Structures were validated using MolProbity [32]. Refinement and model quality statistics are presented in Table 1. Structure figures were prepared in PyMOL [33]. The homodimer-forming interaction surface was calculated using the manganese-bound *Ba* NrdF structure and the PDBsum tool [34].

## Results and discussion

In vitro, class Ib NrdF self-assembles to the Fe^III^/Fe^III^-tyrosyl radical-containing form in the presence of Fe^II^ ions and dioxygen. The Mn-containing NrdF is likely the most relevant form in vivo, with the Mn^III^/Mn^III^-tyrosyl radical as its active state [35–38]. The protein self-assembles to the Mn^II^/Mn^II^ form in the presence of Mn^II^ ions, but does not generate the tyrosyl radical, because it does not react directly with molecular oxygen. If metals are removed, *Ba* NrdF remains stable in vitro and is capable of forming a di-nuclear Fe or Mn cofactor in the presence of the respective divalent metal ions.

Here, we present five structures of class Ib *B. anthracis* NrdF derived from apo protein and loaded with Mn^II^ or Fe^II^ under anoxic or aerobic conditions after crystallization. The crystal structures were determined to high resolution (refer to Table 1 for data quality and refinement statistics). The structures are abbreviated as follows: metal-free *B. anthracis* NrdF—apo *Ba* NrdF, Fe-loaded protein in the presence of oxygen—Fe_2_-aer *Ba* NrdF, Fe-loaded protein in the absence of oxygen—Fe_2_-anox *Ba* NrdF, Mn-loaded protein in the presence of oxygen—Mn_2_*Ba* NrdF, and the structure of Fe-containing *Ba* NrdF prepared in the presence of oxygen and exposed to a highly attenuated X-ray beam—Fe_2_-semiox *Ba* NrdF.

Prior to protein crystallization and metal-soaking experiments, the *Ba* NrdF protein solution was analyzed by TXRF to ensure that it was metal-free. The content of manganese and iron as well as other transition metals (Ni, Cu, and Zn) in the sample was negligible (≤ 0.1 metal per protein). For the structural analysis of *Ba* NrdF in complex with iron or manganese prepared aerobically and anaerobically, crystals of apo protein were soaked with Fe^II^ or Mn^II^ salts (see “Methods” for details).

### Overall structures of *B. anthracis* NrdF

**Fig. 1:**
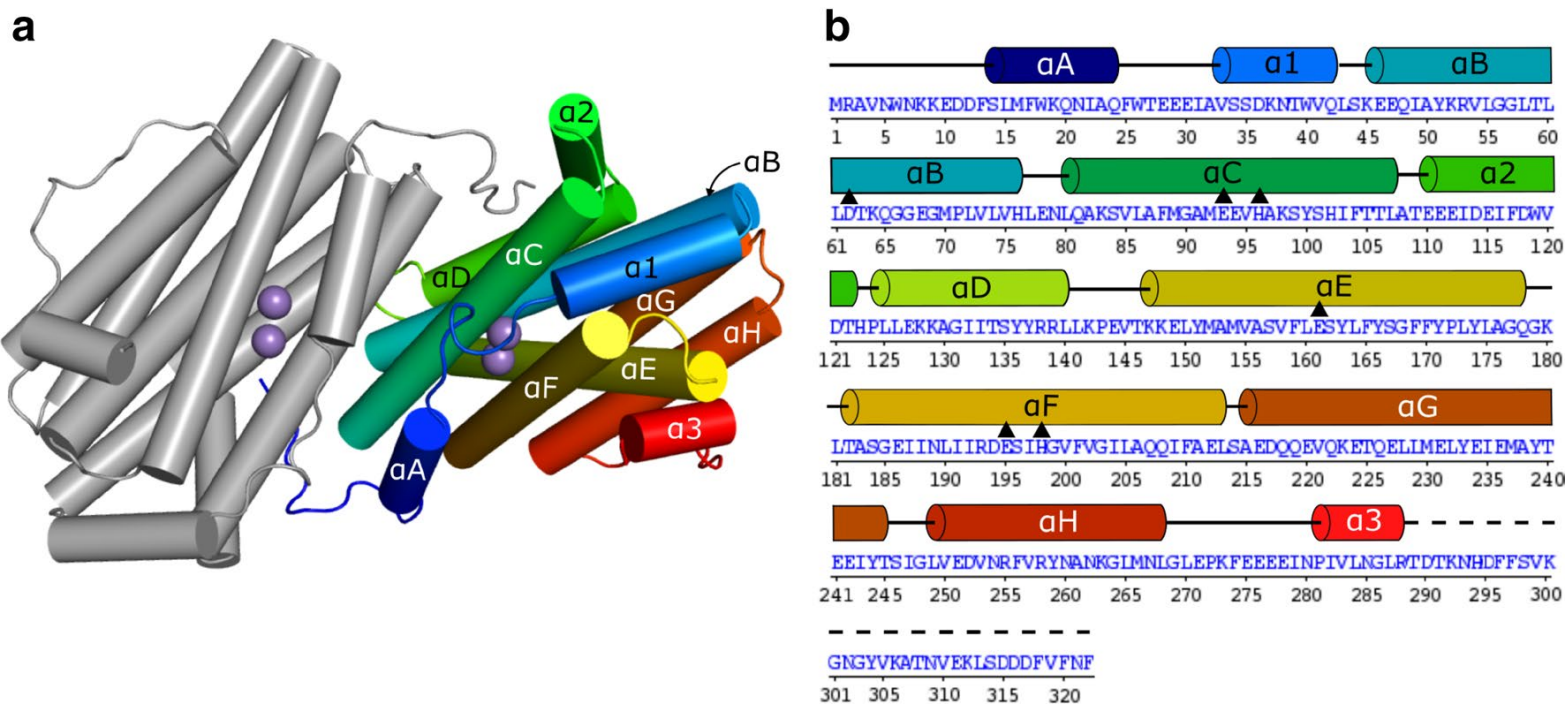
Overall architecture of the *B. anthracis* NrdF homodimer. **a** Cartoon representation of the manganese-bound *Ba* NrdF homodimer. Helices are shown as cylinders and colored in rainbow (blue: N-terminus; red: C-terminus) in one of the protein subunits; the other subunit is shown in grey. The manganese ions in the di-nuclear metal centers are shown as purple spheres. Helix nomenclature is adapted from Ref. [40]. **b** Sequence of *Ba* NrdF with aligned secondary structure. Metal-binding residues are indicated as black triangles. Dashed lines indicate parts of the structure that were not resolved in the electron density

The *Ba* NrdF is as a heart-shaped homodimer with a monomer–monomer interaction surface of about 2200 Å^2^, mostly built by hydrophobic interactions and pronounced exchange of the N-termini between monomers. Each monomer comprises a helix bundle fold similar to other class Ib R2 proteins [39] (Fig. 1). All structures of *Ba* NrdF show well-defined electron density, except for the flexible poly-glutamate region (E275–E278) in the loop connecting helices αH and α3. The last 34 residues of the C-terminus, important for interaction with the catalytic subunit R1 [39], are disordered, with the exception of apo and Fe_2_-aer *Ba* NrdF, where seven extra residues of the C-terminus form an additional small helix. Globally, the apo *Ba*NrdF and the Fe- or Mn-containing forms are almost identical, with the largest RMSD value of 0.25 Å over all C_α_ atoms, compared to apo *Ba* NrdF. Interestingly, the structures reveal two proline residues, unusually located within a helix while not breaking the helical secondary structure. The first proline residue, P71, is located on αB at the core of a dimer interface. The second proline residue, P172, is localized on αE in the vicinity of the conserved metal-binding site.

### Structures of the apo and metal-loaded *B. anthracis* NrdF metal sites

**Fig. 2:**
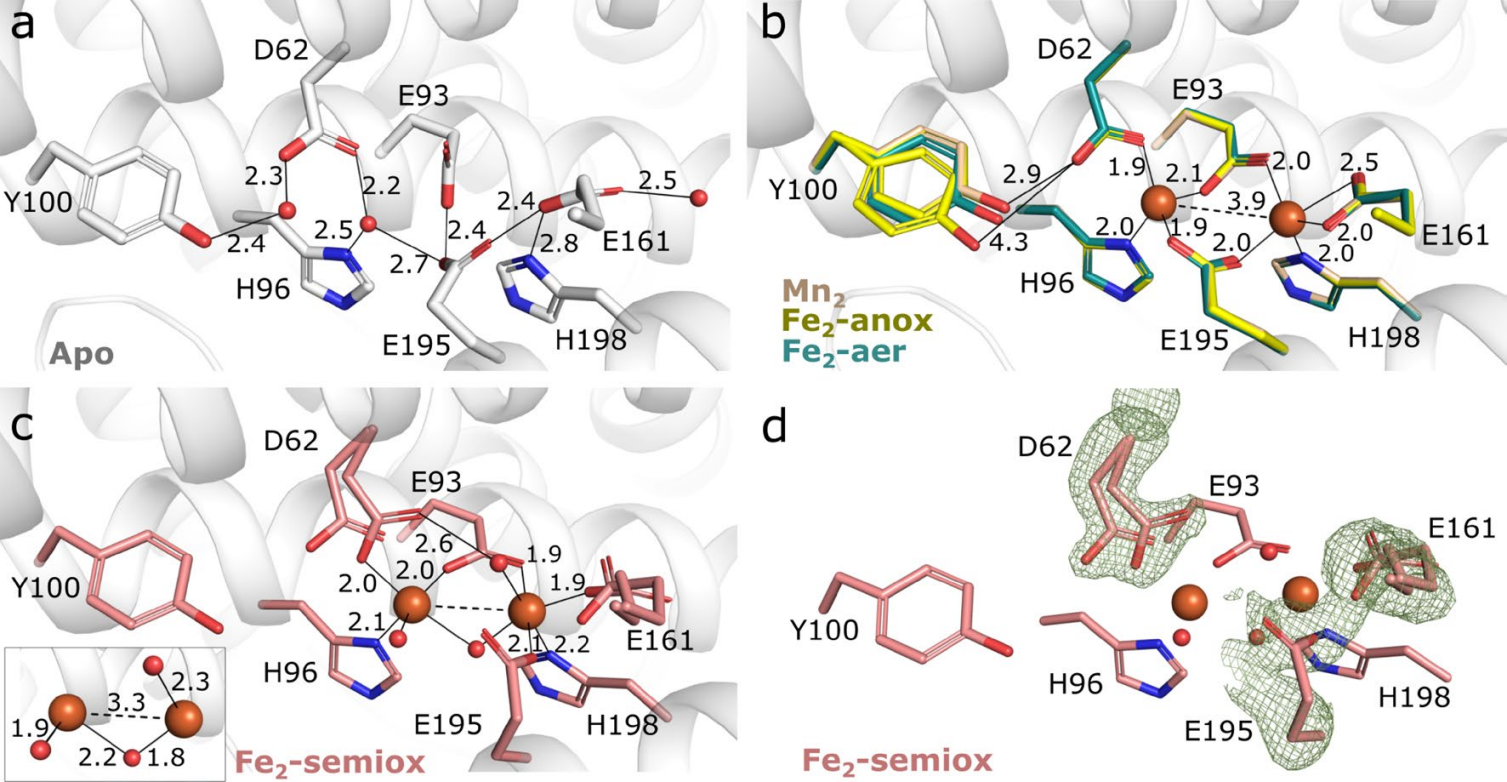
Coordination environment of *Ba* NrdF metal sites in different metalation and oxidation states. **a** Metal site of the apo *Ba* NrdF. **b** Overlay of the Fe_2_-aer (green), Fe_2_-anox (yellow) and Mn_2_*Ba* NrdF (wheat) metal sites representing reduced states. The positions of manganese (II) and ferrous ions as well as Y100 in Mn_2_ and Fe_2_-anox structures superpose perfectly. A low occupancy solvent molecule coordinating Y100 was omitted for clarity. **c** Metal-chelating ligands D62, E161, and E195 in the oxygen-activated Fe_2_-semiox structure represent a transition from oxidized to reduced coordination modes. The insert shows the aqua-bridged di-ferric metallo-cofactor with an additional water molecule coordinating each metal ion. **d** Omit *F*_o_–*F*_c_ electron density map (green mesh) around D62, E161, and E195 in Fe_2_-semiox structure illustrating their conformational flexibility. The map is contoured at 3.5σ and shown within a 1.6 Å radius from these residues. Solid lines indicate hydrogen or ionic bonds and dashed lines indicate metal–metal distances (in Å). Water molecules are shown as small spheres and metal ions are shown as large spheres

The metal-binding site of each subunit of *Ba* NrdF consists of two histidines (H96 and H198) and four carboxylate ligands conserved in class Ia and Ib R2 proteins (D62, E93, E161, and E195), located in the core of the helix bundle of each protomer on helices αB, αC, αE, and αF (Fig. 1a, b). Notably, in class Ic and Id orthologs, the first carboxylate (D62 in *Ba*) is a glutamic acid, whereas in the newly discovered class Ie, three of the carboxylates are substituted, precluding metal binding [4, 5, 8]. Structures of the *Ba* NrdF metal sites are shown in Fig. 2a–d.

In the apo structure, a water molecule, hydrogen bonded to D62, H96 and E195, occupies metal site 1, proximal to the radical-carrying residue Y100. Metal site 2, in turn, is vacant, with E161, H198, and E195 forming an H-bond network. Notably, the conformation of E195 seems to be stabilized by hydrogen bonds with E93 and E161, resulting in very short H-bond distances between these residues, refining to 2.4 Å. In addition, E161 is hydrogen bonded to a water molecule (Fig. 2a).

The structures of the reduced metal-binding sites in Fe_2_-anox and Mn_2_-bound *Ba* NrdF are identical within experimental error in terms of the metal ligand conformation and hydrogen-bonding pattern: the metal ions in sites 1 and 2 are 4- and 5-coordinated, respectively, with a typical bond length of 1.9–2 Å (Fig. 2b). The metal in site 1 has three monodentate carboxylate ligands: D62, E93, and E195, and an additional bond to H96. The metal in site 2 also has two monodentate carboxylate ligands, E93 and E195, and an additional bond to H198. E161 coordinates the metal ion in site 2 in a bidentate fashion; however, one of the carboxylate-to-metal distances is somewhat longer. Despite the expected metallo-cofactor activation by oxygen in the Fe_2_-aer crystal, there is no evidence of a µ-aqua, hydroxo, or oxo bridge, and the metal site seems to represent a reduced state, as judged by the conformation of the metal-coordinating ligands, the metal–metal distance (3.9 Å), and the fact that the structure of the metal site is within experimental error identical to the reduced di-manganese and Fe_2_-anox (di-ferrous) structures (Fig. 2b). Given the high sensitivity of R2 proteins to X-ray photo-reduction [41], a property that appears particularly pronounced in class Ib proteins [25, 42, 43], the metallo-cofactor of the Fe_2_-aer structure was most likely photo-reduced during X-ray data collection. Interestingly, the redox-active Y100 side chain displays a dual conformation. It is particularly well defined in both fully reduced structures—Fe_2_-anox and Mn_2_*Ba* NrdF, and to a lesser extent in the Fe_2_-aer structure. To our knowledge, this feature has not been previously reported for class Ib R2 proteins (discussed further below).

**Fig. 3:**
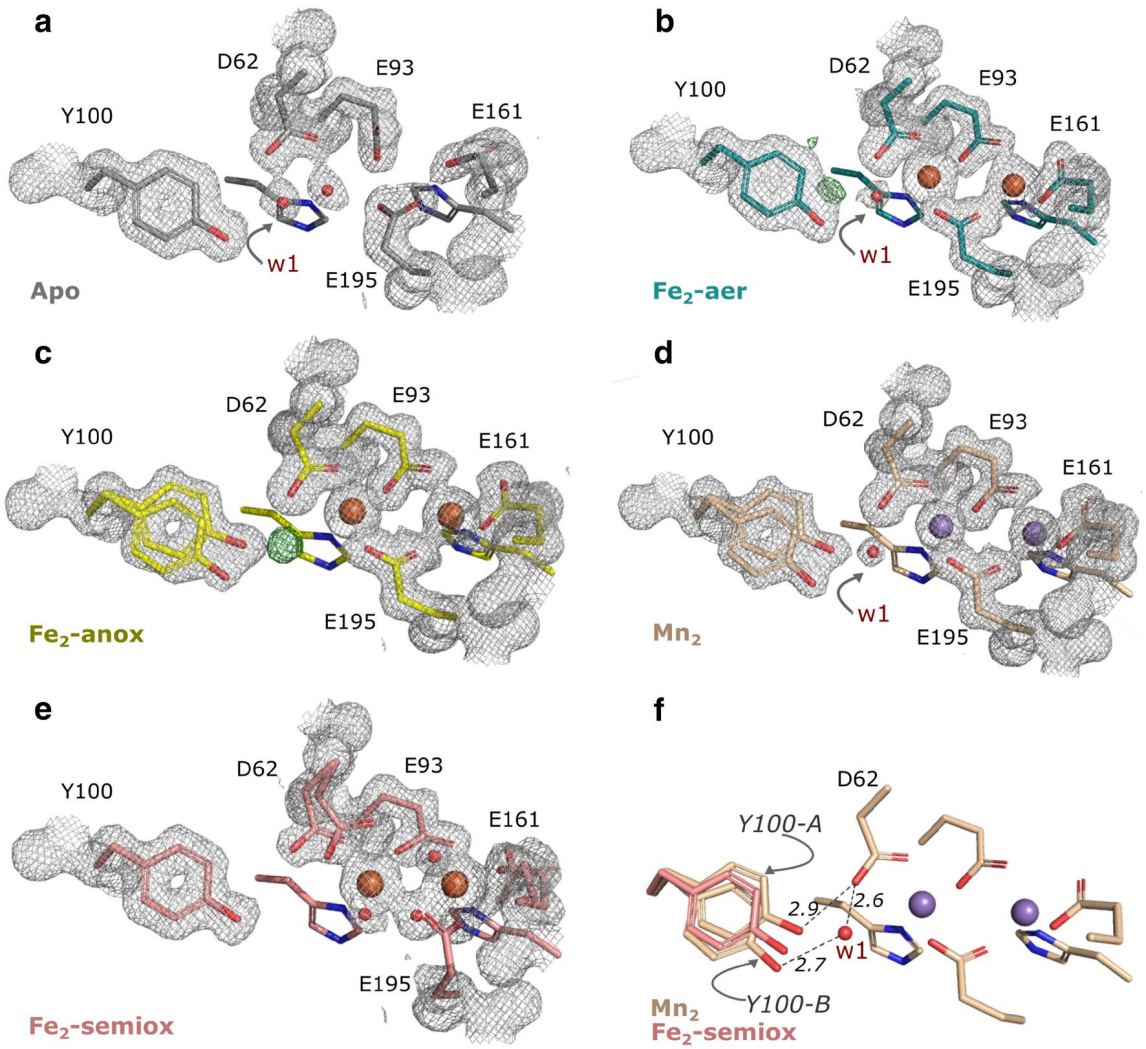
Conformational dynamics of the redox-active tyrosine in Ba NrdF structures. **a** Metal site of the apo Ba NrdF. The Y100-B conformation is stabilized by a w1-mediated hydrogen bond to D62. **b** Metal site of the Fe_2_-aer Ba NrdF in chain B. The Y100-B conformation is stabilized by a w1-mediated hydrogen bond to D62. The positive difference electron density map (green mesh) indicates that the alternative, Y100-A conformation is possible. **c** Metal site of the Fe_2_-anox Ba NrdF. The positive difference electron density map (green mesh) corresponds to solvent molecule w1. Y100 is modeled in two alternative conformations. **d** Metal site of the Mn_2_ Ba NrdF. Y100 is modeled in two alternative conformations. The Y100-B conformation is stabilized by a w1-mediated hydrogen bond to D62. The hydroxyl group of Y100-A clashes with w1. **e** Metal site of the Fe_2_-semiox Ba NrdF. The position of Y100 refines as an average conformation between Y100-A and Y100-B. **f** Positional overlay of Y100 in Fe_2_-semiox and Mn_2_ Ba NrdF metal sites with highlighted differences between Y100-A and Y100-B conformations and the role of w1 in stabilization of the conformation of Y100-B. Dashed lines indicate hydrogen bond distances (in Å). The density map for H96 and H198 is not shown for clarity. The 2*F*_o_–*F*_c_ electron density map is contoured at 1.2σ and shown as a grey mesh. The *F*_o_–*F*_c_ positive difference electron density map (green mesh) around Y100 is contoured at 3.2σ and shown within a 1.6 Å radius from the residue. Water/oxo/hydroxo ligands are shown as red spheres. Metal ions are shown as larger spheres: Fe—in orange and Mn—in purple

Finally, we attempted to obtain a structure of *Ba* NrdF with an oxidized metallo-cofactor. Apo *Ba* NrdF crystals were soaked with ferrous salt in the presence of oxygen and exposed to a highly attenuated X-ray beam for data collection to decrease the effect of photo-reduction. The resulting structure (Fe_2_-semiox) mainly displays structural features of the oxidized metallo-cofactor in both chains of the dimer, although it is obvious that photo-reduction was not completely avoided. Compared to the fully (photo-) reduced *Ba* NrdF structures, the metal ions in both metal centers of the Fe_2_-semiox structure are linked by a monoatomic bridge, *cis* side relative to the metal-coordinating histidines, and the metal–metal distance is decreased to 3.3 Å. In addition, one solvent molecule coordinates to each Fe ion. Upon oxidation, the coordination pattern of the carboxylate ligands also changes. D62 is still monodentate to Fe in site 1, yet further away from Y100 compared to the reduced state. E161 and E195, on the other hand, appear to switch to a monodentate coordination to the Fe ion in site 2, as evident from the shape of the *F*_o_–*F*_c_ omit difference electron density (Fig. 3c). E93 remains in a bidentate coordination mode, bridging both metal ions. Despite the partial occupancy of a reduced site, the oxidized structure could be modeled. In the oxidized structure, the Fe ion in site 1 is five-coordinate, while the ion in site 2 is six-coordinate. The structure thus appears similar to the di-ferric *Escherichia coli* (*Ec*) class Ia NrdB and di-manganese (III) *Corynebacterium ammoniagenes* (*Ca*) NrdF in the resting (met) state [35, 44].

### Conformational variation of the redox-active tyrosine residue in *B. anthracis* NrdF structures

Compared to the apo *Ba* NrdF, the metal-containing structures display a movement of the redox-active Y100 relative to D62 and metal site 1 (Fig. 3a–f). Y100 in Fe_2_-aer, Fe_2_-anox, and Mn_2_-bound *Ba* NrdF exists in a dual conformation (Y100-A and Y100-B). Previously reported high-field electron paramagnetic resonance (EPR) spectroscopy data on Fe-containing NrdF proteins from *Salmonella typhimurium* (*St*), *Ba* and *Ec* class Ia NrdB indicated a shift of the tyrosine (Y105 in *St* and Y122 in *Ec*) as a result of its oxidation to Y· [15, 44, 45], resembling the conformational switch from Y100-A to Y100-B in our *Ba* NrdF structures (Fig. 3). However, given that Fe_2_-aer, Fe_2_-anox, and Mn_2_-bound *Ba* NrdF clearly represent reduced states of the metallo-cofactors, attribution of the conformational change to Y· formation in this case appears implausible. Still, it is interesting to note that the tyrosine in *Ba* NrdF exhibits conformational dynamics that may contribute to radical stabilization and initiation of radical transfer.

The Y100-B conformation appears to be stabilized by an adjacent water molecule (*w1*) found in the apo, Fe_2_-aer and Mn_2_-bound *Ba* NrdF structures (Figs. 2a,  3a–d). The *w1* molecule bridges Y100 and D62 (Fig. 3f). In the Fe_2_-anox metallo-cofactor site, the *w1* is present at a low occupancy, as judged by the characteristic positive difference density peak in proximity to the split Y100 hydroxyls. The low occupancy of *w1* also appears to correlate with the occupancy of the Y100-B conformer in this structure (Fig. 3c). In the oxygen-activated Fe_2_-semiox structure Y100 refines best, within experimental error, as an average conformation between Y100-A and Y100-B (Fig. 3e, f) and no positive electron density corresponding to low-occupancy *w1* can be observed. This conformational state of Y100 is potentially associated with subsequent decoupling from the metal site associated with the redox state-dependent shift of D62.

### Distorted topology of αE at the metal site

**Fig. 4:**
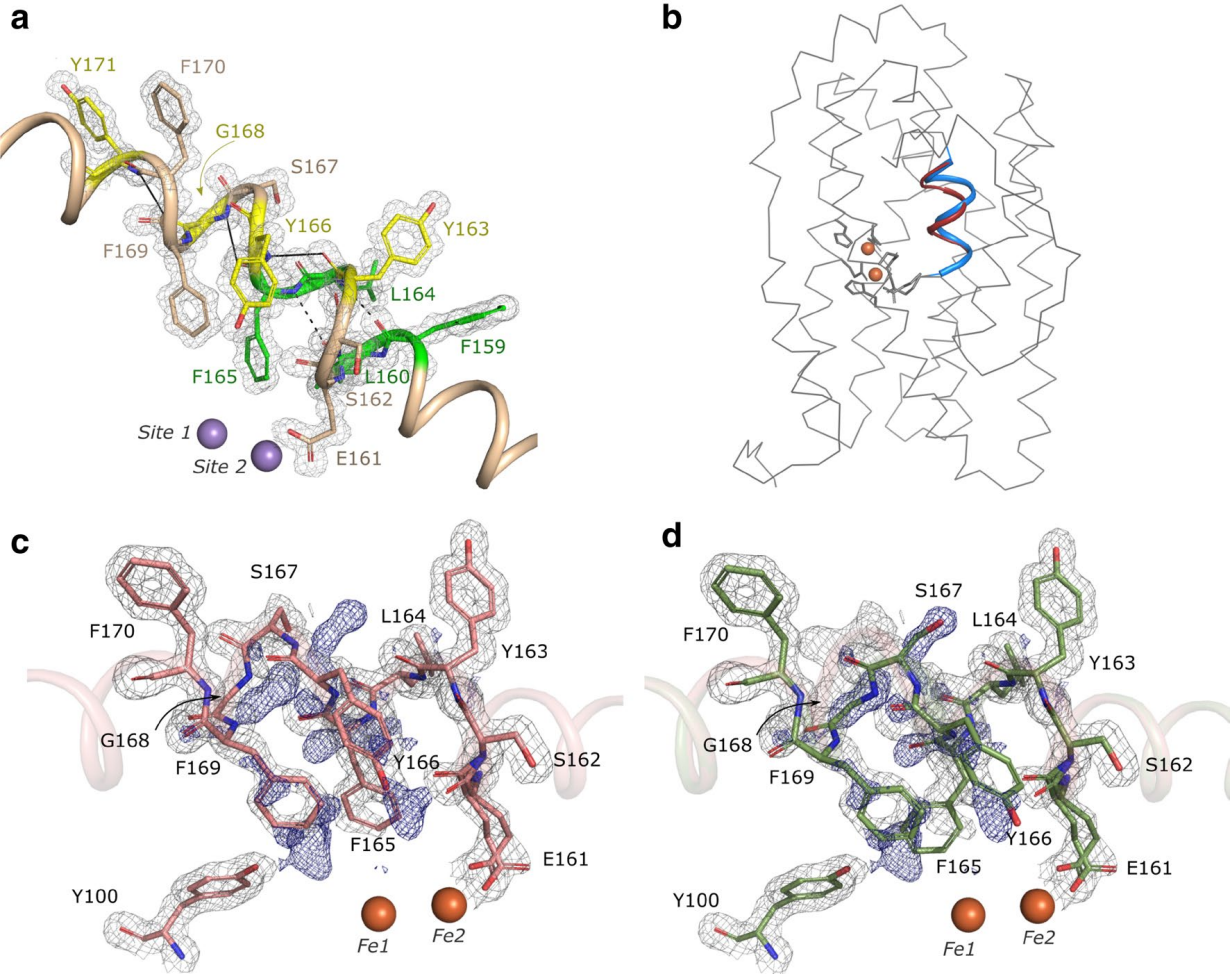
Helix E undergoes structural rearrangements upon metallo-cofactor oxidation. **a** Residues participating in *i* + 5 interactions are shown in green and the respective hydrogen bonds are shown as dashed black lines. The residues participating in 3_10_-type (*i* + 3) interactions are shown in yellow and the respective hydrogen bonds are shown as solid black lines. Metal ions (Me1 and Me2) are shown as spheres. **b** Cartoon illustration of conformational differences in αE upon metallo-cofactor oxidation in the *Ba* Fe_2_-semiox structure. The αE conformation corresponding to the reduced state is highlighted in blue, oxidized—in red. Chain B of the *Ba* NrdF heterodimer is hidden for clarity. **c** αE kink in *Ba* Fe_2_-semiox is modeled as in (photo-) reduced structures; however, positive difference electron density (shown as blue mesh) suggests structural rearrangements upon oxidation. **d** Model of αE rearrangements upon metallo-cofactor oxidation, as suggested by positive *F*_o_–*F*_c_ electron density. The 2*F*_o_–*F*_c_ electron density map is contoured at 1.2σ and shown as a grey mesh within 1.2 Å radius from the residues. The *F*_o_–*F*_c_ electron density map is contoured at 2.5σ and shown as a blue mesh within 2.0 Å radius from the residues

αE is one of the helices providing one of the six metal-chelating ligands in R2 proteins, namely, E161 in *Ba* NrdF. In all our structures, as well as all other NrdF protein structures published to date, αE exhibits rather unusual distorted topology in close proximity to the metal site; within amino-acid stretch F159–Y171 (*Ba* numbering). This region has both π-helical and 3_10_-helical features. As in all π-helices, the standard intra-helical hydrogen-bonding pattern *i* + 4, characteristic for α-helices, is disrupted by amino-acid insertions, causing a switch to *i* + 5 (carbonyl of L160 to amide proton of F165 and carbonyl of F159 to amide proton of L164, as shown in Fig. 4a). In addition, a number of main chain carbonyl groups within αE are not intra-helically hydrogen bonded. Particularly, the carbonyl group of E161, which participates in metal coordination of site 2 and was shown to be relatively flexible during metallo-cofactor oxidation-state changes, is hydrogen bonded to the Y166 hydroxyl, bridged by a solvent molecule. This is in agreement with what has been previously observed in *B. subtilis* class Ib R2 [46]. Similarly, through a water-mediated hydrogen bond, the main chain carbonyl group of S162 is linked to N260 and the carbonyl group of L164 is linked to E236. Finally, the main chain carbonyl of S167 directly interacts with Y244.

Several residues of αE are hydrogen-bonded in an *i* + 3 pattern, characteristic of 3_10_-type helices. These somewhat unusual hydrogen bonds occur between the carbonyl groups and amide protons, respectively, in the following residue pairs: Y163–Y166, F165–G168, and G168–Y171 (Fig. 4a). The intra-helical hydrogen-bonding pattern within the F159–Y171 amino-acid stretch does not change upon metal binding (specifically in the reduced and photo-reduced *Ba* NrdF structures) irrespective of the metal type bound.

### Structural rearrangements in αE upon oxidation of the di-iron metallo-cofactor in *B. anthracis* NrdF

π-helices are often found close to protein functional sites and are energetically expensive [47]. This somewhat distorted region of the helix corresponding to αE in *Ba* NrdF is conserved in many ferritin superfamily members: class I R2 proteins, R2-like ligand-binding oxidases (R2lox), bacterial multicomponent monooxygenases (BMMs), and aldehyde-deformylating oxygenases (ADO) among others, suggesting a functional role [48, 49]. The purpose of this high-energy conformation throughout the superfamily, however, is enigmatic.

The F159–Y171 helix segment in the Fe_2_-semiox structure undergoes conformational changes compared to the apo and reduced *Ba* NrdF structures, as evident from the positive difference *F*_o_–*F*_c_ maps (Fig. 4b–d). The dominant conformation of the F159–Y171 helix segment in the Fe_2_-semiox *Ba* NrdF is identical to that in the (photo-) reduced *Ba* NrdF structures (Fig. 4a, c). To visualize the conformational changes and assess their possible role in protein function, we used the positive difference *F*_o_–*F*_c_ density map for building the alternative conformation of the αE main chain and side chains, where possible (Fig. 4c, d; Supplementary Movie 1). Notably, the alternative conformation of the helix (Fig. 4e) is very similar to one reported by Cox et al. for the oxidized di-manganese *Ca* NrdF (PDB ID: 3MJO) [35] (Supplementary Movie 2). The most dramatic conformational changes were observed for residues F165, Y166, and S167, all located on the apical side of the metal site. Particularly, S167 forms a new intra-helical hydrogen bond with the F170 backbone amide while losing a bond with the E236 carboxyl (located on αG) and causes Y166 to slide out significantly towards the αF main chain. The F165 side chain capping the metal site flips and stacks against F169 and the radical-generating Y100. Similar conformational changes in αE have been previously described for Fe-containing *St* NrdF. Ericsson et al. propose a relation between the alternate conformation of αE and metallo-cofactor oxidation states [50]. Cotruvo et al. [10] propose that αE may control a joint event of superoxide delivery from NrdI to NrdF and then trigger NrdI dissociation from the complex once the process is complete.

The timing of the conformational change of the αE backbone is elusive. It is possible that the structural rearrangements are initiated by a carboxylate shift of E195 in response to metallo-cofactor activation. However, it cannot be excluded that this process is triggered earlier by the superoxide interaction with the hydrophilic channel [10] or the water molecule stabilizing the reduced π-helix conformation [46]. Once E195 flips and changes to monodentate coordination to the Fe ion in site 2 (Fig. 2b–d), its liberated oxygen stacks against the phenyl ring of F165. Consequently, the F165 side chain flips, as evident from the incomplete *2F*_o_–*F*_c_ electron density around the F165 phenyl ring (Supplementary Fig. 1a). Once flipped, F165 then stacks against the strictly conserved F169, possibly forming aromatic interactions between the phenyl rings. Finally, the F169 side chain, together with F165, closes the hydrophobic pocket around Y100 from the apical side of the metal site.

The residue in position 169 (*Ba*) is strictly conserved as phenylalanine in R2 proteins (F166 in *Ec* and *St*; F176 in *Ca*, F169 in *Bacillus cereus* (*Bc*) and even in the newly discovered class Ie NrdF proteins [4, 5]) and may play an important role in shielding the radical site, thus contributing to its stability and potentially mobilization for delivery to protein R1. The side chain corresponding to F165, on the other hand, is conserved as phenylalanine or leucine in R2 proteins and caps the metal site on the side, where the reaction with oxidant is expected to take place. It appears plausible that this residue is involved in regulation of electron transfer between the redox-active tyrosine Y100 and the metal site. In addition, the F165–F169 side chain pair may be involved in preventing radical loss by closing the hydrophobic pocket around Y100. Interestingly, the Mn^II^-bound *Bc* and *Ec* NrdF structures also display rotation of the side chain equivalent to F165 in *Ba* (F165 in *Bc* and F162 in *Ec*). Here, the phenylalanine flips to make space for the glutamate, corresponding to E161 in *Ba* (E161 in *Bc* and E158 in *Ec*), which coordinates to both Mn ions on the *trans* side relative to the metal-coordinating histidine residues [12, 51]. However, no alternate conformations of αE can be observed.

### The hydrophilic NrdI–NrdF channel is closed when the metallo-cofactor is oxidized

**Fig. 5:**
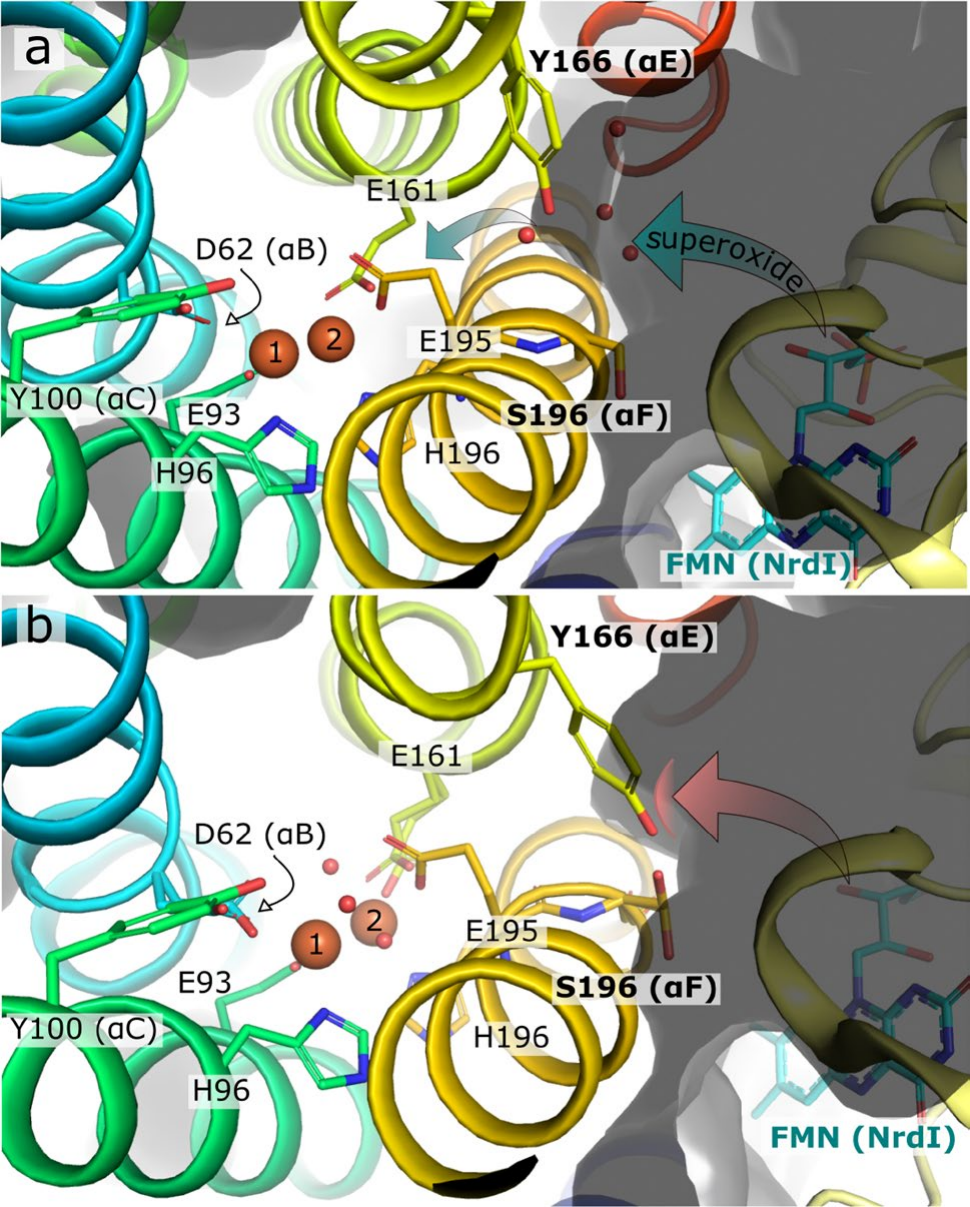
Proposed gating of the NrdI–NrdF (FMN–metal cluster) solvent/oxidant channel. **a** Docked model of the FMN-bound NrdI and Ba Fe_2_-semiox NrdF (based on the Bc complex, PDB id: 4BMO). A surface-slice view of the open solvent/superoxide channel (blue arrows), connecting the FMN cofactor and the metal cluster (modeled as reduced) of the Ba NrdF. **b** Upon oxidation of the metal cluster, the distorted αE segment rearranges; Y166 seals the solvent/superoxide channel (red arrow) by making a polar interaction with αF. The helices of Ba NrdF are colored, as shown in Fig. 1. Metal ions are shown as large orange spheres, FMN in cyan sticks. Residues gating the solvent access channel are labeled in bold. The protein surface is shown in translucent grey

Class Ib R2 s have been shown to function as di-manganese proteins in vivo [35–38]. A flavin mononucleotide (FMN)-containing activase, denoted NrdI and conserved in class Ib and Ie R2 proteins, was found to be essential for Mn-ion oxidation and subsequent Y· generation, since Mn^II^ generally does not react spontaneously with molecular oxygen [10]. The NrdF–NrdI complex forms a channel, connecting the FMN cofactor in NrdI with the metal site in NrdF, presumably utilized for delivery of the oxidant species to the Mn^II^/Mn^II^ cofactor [11–13]. NrdF–NrdI complex crystal structures are available for the orthologs from *E. coli* (di-manganese form) [12] and *B. cereus* (di-iron form) [51] with reduced metallo-cofactors in both structures. These NrdF proteins are 42% and 99% identical in sequence to *Ba* NrdF, respectively. Given the very high sequence similarity with *Bc* NrdF, the interaction surface with the NrdI and the FMN in *Ba* NrdF can be presumed to be the same. Therefore, we docked the *Bc* NrdI and *Ba* Fe_2_-semiox NrdF. In the docking model, the likely solvent passage linking the FMN and the metal in site 2 in NrdF extends between helices αE and αF (Fig. 5). In addition, we compared our *Ba* NrdF–NrdI model to the crystallographically characterized native *Ec* NrdF–NrdI complex [12].

The Fe_2_-semiox *Ba* NrdF electron density data, together with data reported by Cox et al. for oxidized di-manganese *Ca* NrdF, suggest that orchestrated conformational changes in the NrdF subunit are triggered by reaction with oxygen. Particularly, the carboxylate shift of E195 synchronized with changes in metal–metal distance and solvation cause choreographed movement of the αE backbone in the NrdF subunit, likely initiated at the aromatic side chains (F165 and F169). The phenylalanine side chains flip and seal the hydrophobic pocket around the Y100 radical from the metallo-cofactor side. At the same time, adjustments of the aromatic residues cause significant intra-helical hydrogen bond rearrangements in the αE π-/3_10_-helical region (Fig. 4), forcing Y166 to move towards αF (at residue S196). It is not unlikely that Y166 and S196 (Y163–A193 in *Ec* and *St*, Y173-S203 in *Ca* NrdF) form a hydrogen bond, and the solvent passage is then sealed (Fig. 5 and Supplementary Movies 1 and 2). In fact, this hydrogen bond is evident in the oxidized di-iron *St* and di-manganese *Ca* NrdF (Ref. [35, 50]). In the absence of NrdI, Y166 is exposed to disordered surface solvent in all NrdF structures published to date; its hydroxyl-to αF distance, therefore, varies. The side chain corresponding to Y166 in *Ba*, together with other polar residues that line the solvent passage from the FMN to metal site 2, is conserved [12, 51].

In *Ec* NrdF complexed with NrdI (PDB ID: 3N39 and 3N3A), the solvent passage to the di-manganese metal appears to be open, which is in good agreement with the metallo-cofactor oxidation state in these crystal structures [12]. In Mn-loaded *Ec* NrdF, the aromatic side chain corresponding to F165 in *Ba* adopts an unusual conformation (F162 in *Ec*), similar to the one in the oxidized *St* and *Ca* structures [35, 50] and resembling the proposed alternative conformation in Fe_2_-semiox *Ba* NrdF (Fig. 4c, d). However, in the Mn-loaded *Ec,* the conformation of the F162 side chain is not oxidation-state dependent, but rather metal-ion-type dependent, since Fe-loaded *Ec* NrdF (PDB ID: 3N38, Ref. [12]) resembles the (photo-)reduced structures of *Ba* NrdF. Apart from the metal site proximal phenylalanine pair, a glutamate residue equivalent to E161 in *Ba* and located on αE was proposed to regulate access to the metal site [12, 51]. Therefore, it is possible that there are other oxidation-state-sensing residues involved in locking the NrdF–NrdI superoxide passage in NrdF proteins. The structural data thus suggest that the passage for the hydrophilic oxidant species from the FMN cofactor in the NrdI to the metal site in the NrdF is closed once the metal site is oxidized and the Y· is generated. This could prevent premature loss of the radical to solvent and also stop the injection of a second superoxide species, which would likely rapidly quench the tyrosyl radical.

## Conclusion

In the present study, we report several crystal structures of *B. anthracis* NrdF—the radical-generating subunit of class Ib RNR. These high-resolution structures allow a detailed analysis of the metallo-cofactor environment and the radical site in the presence of Mn/Mn or Fe/Fe sites, as well as in the metal-free form. In contrast to Mn-containing NrdF (most relevant in vivo, Ref. [35–38]), the di-ferrous metallo-cofactor spontaneously reduces oxygen and forms a di-ferric metallo-cofactor and the Y· in vitro, and possibly in vivo, depending on metal-ion availability. As for the other class Ib NrdF proteins characterized to date, *Ba* NrdF is extremely sensitive to X-ray photo-reduction. Nevertheless, we were able to determine the structure of oxygen-activated di-Fe NrdF by exposing a crystal to very low intensity X-rays.

Upon metal-ion oxidation and radical generation, NrdF must effectively initiate radical transfer and protect the radical from premature decay or loss to solvent. The crystal structures reported here exhibit interesting features, likely to be functionally relevant. First, our structures reveal that in *Ba* NrdF, the redox-active tyrosine side chain (Y100) displays conformational flexibility, irrespective of the type of metal-ion bound. These dynamics may be important in radical generation and transfer initiation to the catalytic subunit of RNR, R1. Second, the oxidized di-Fe structure (Fe_2_-semiox *Ba* NrdF) reveals significant orchestrated conformational changes in the primary and secondary coordination spheres of the metal ions, compared to reduced and photo-reduced *Ba* NrdF. Particularly, a conserved π-/3_10_-helical segment of αE, located proximally to the metal site undergoes significant rearrangements, following the oxygen activation. We hypothesize that these structural rearrangements play a central role in sealing the solvent channel, linking the FMN cofactor of NrdI and the metal site in NrdF, and serving as a superoxide passage necessary for di-Mn metallo-cofactor activation in vivo [11–13]. Comparable mechanisms of cofactor shielding have been shown for, e.g., nitrogenase and [FeFe]-hydrogenase, and proposed to be important for correct cofactor assembly [52]. In addition, this π-/3_10_-helical segment of αE may play a role in insulating the hydrophobic pocket around the Y· by stacking the strictly conserved Phe169 side chain against the redox-active tyrosine side chain Y100. Together, these findings further emphasize the relevance of structural dynamics in R2 proteins and expand our understanding of radical generation and shielding in RNRs and may be useful for structure-based drug design against anthrax.

## Electronic supplementary material

Below is the link to the electronic supplementary material.

Supplementary material 1 (PDF 562 kb)

Supplementary material 2 (MPG 779 kb)

Supplementary material 3 (MPG 14010 kb)

## Abbreviations

ADO: Aldehyde-deformylating oxygenase
BMM: Bacterial multicomponent monooxygenase
EPR: Electron paramagnetic resonance spectroscopy
PDB: Protein data bank
FMN: Flavin mononucleotide
R1: Large subunit of ribonucleotide reductase, also called *α*
R2: Radical-generating subunit of ribonucleotide reductase, also called *β*
R2lox: R2-like ligand-binding oxidase
RNR: Ribonucleotide reductase
TXRF: Total-reflection X-ray fluorescence
