# Supplementary material for: Redox-induced structural changes in the di-iron and di-manganese forms of Bacillus anthracis ribonucleotide reductase subunit NrdF suggest a mechanism for gating of radical access
Source: J Biol Inorg Chem. 2019 Aug 13;24(6):849–61. doi: 10.1007/s00775-019-01703-z (PMC6754363; doi:10.1007/s00775-019-01703-z)
Supplement: Supplementary file 1 — Supplementary material 1 (PDF 562 kb) [file 775_2019_1703_MOESM1_ESM.pdf]

Supplementary information

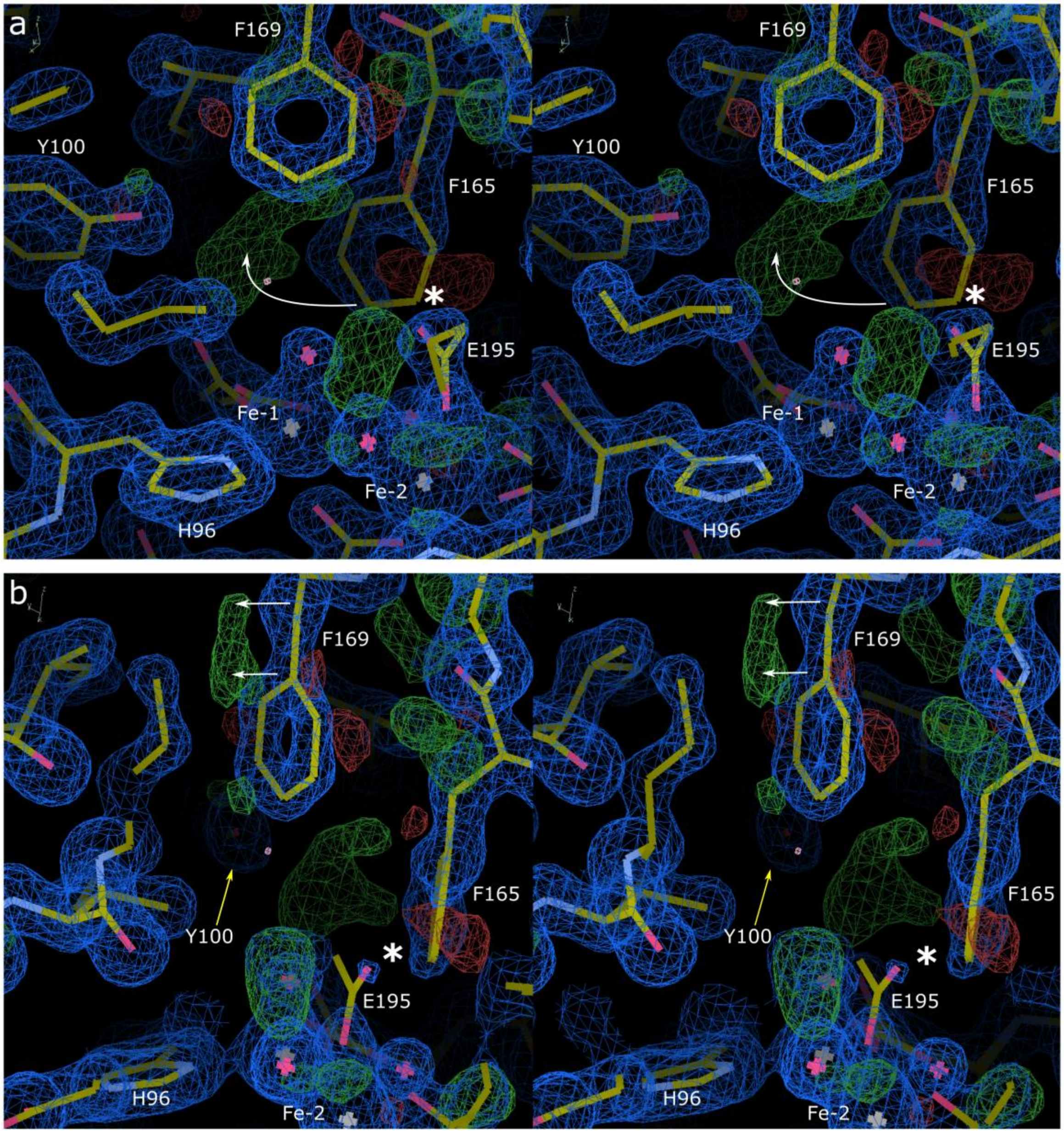

Supplementary figure 1. Wall eyed stereo images of the electron density illustrating conformational flexibility of the aromatic side chains (on αE) at the metal site upon oxygen activation.

(a) *A close up view of the metal site in Fe_2-semiox Ba NrdF structure, illustrating an alternative conformation of F165, proximal to E195 coordinating the metal ion in site 2.* White arrow indicates flip

of F165 side chain. ***(b)*** *A close up view of the metal site in Fe_2-semiox Ba NrdF structure at a different angle, illustrating an alternative conformation of F169.* White arrows indicate F169 side chain shift. *The F_o-F_c difference electron density map is contoured at 3 σ (positive difference density – green mesh, negative – red mesh). The $2F_o$-F_c electron density map is shown as blue mesh and contoured at 1.6 σ.* White star indicates clash between E161 oxygen atom and phenyl ring of F165.

Supplementary Movie 1.

A morph between the dominant/reduced conformation of an αE π-helical region (in salmon cartoon/sticks) and its alternate/oxidized conformation (in green cartoon/sticks) observed in Fe_2-semiox *B. anthracis* NrdF structure. Fe-ions are shown as spheres. Solvent molecules are omitted for clarity.

Supplementary Movie 2.

Overlay of an αE π-helical region in *B. anthracis* Fe_2-semiox NrdF (dominant/ reduced conformation – in green sticks; secondary/oxidized conformation in purple sticks) and *C. ammoniagenes* oxidized di-manganese NrdF structures (PDB id: 3MJO, in yellow sticks). Different oxidation states of the metallo-cofactor induce large conformational shift in αE main chain and cause rearrangements in the second metal site coordination shell.

B. anthracis protein residues are labeled in green/purple and *C. ammoniagenes* – in yellow. Metal ions are shown as spheres.
